# Enhancing anaerobic digestion of lignocellulosic biomass by mechanical cotreatment

**DOI:** 10.1186/s13068-024-02521-5

**Published:** 2024-06-03

**Authors:** Anahita Bharadwaj, Evert K. Holwerda, John M. Regan, Lee R. Lynd, Tom L. Richard

**Affiliations:** 1 The Department of Agricultural and Biological Engineering, The Pennsylvania State University, University Park, PA 16802 USA; 2 Thayer School of Engineering, Dartmouth College, Hanover, NH 03755 USA; 3 The Department of Civil and Environmental Engineering, The Pennsylvania State University, University Park, PA 16802 USA

**Keywords:** Anaerobic digestion, Biomethane, Lignocellulose, Cotreatment, Milling, Biogas, Cellulosic, Biofuel

## Abstract

**Background:**

The aim of this study was to increase the accessibility and accelerate the breakdown of lignocellulosic biomass to methane in an anaerobic fermentation system by mechanical *cotreatment*: milling during fermentation, as an alternative to conventional *pre*treatment prior to biological deconstruction. Effluent from a mesophilic anaerobic digester running with unpretreated senescent switchgrass as the predominant carbon source was collected and subjected to ball milling for 0.5, 2, 5 and 10 min. Following this, a batch fermentation test was conducted with this material in triplicate for an additional 18 days with unmilled effluent as the ‘status quo’ control.

**Results:**

The results indicate 0.5 – 10 min of cotreatment increased sugar solubilization by 5– 13% when compared to the unmilled control, with greater solubilization correlated with increased milling duration. Biogas concentrations ranged from 44% to 55.5% methane with the balance carbon dioxide. The total biogas production was statistically higher than the unmilled control for all treatments with 2 or more minutes of milling (α = 0.1). Cotreatment also decreased mean particle size. Energy consumption measurements of a lab-scale mill indicate that longer durations of milling offer diminishing benefits with respect to additional methane production.

**Conclusions:**

Cotreatment in anaerobic digestion systems, as demonstrated in this study, provides an alternative approach to conventional pretreatments to increase biogas production from lignocellulosic grassy material.

**Supplementary Information:**

The online version contains supplementary material available at 10.1186/s13068-024-02521-5.

## Background

Lignocellulosic biomass is abundant and inexpensive but can be difficult to convert into products due to its complex structure. The structure of plant cell walls includes sugar polymers like cellulose and hemicellulose embedded in a hydrophobic lignin matrix, which provides considerable structural strength and resistance to biological attack and disease [1–4]. The resulting cell walls are recalcitrant to deconstruction, which is arguably one of the greatest technical challenges for producing biofuels and biochemicals. Recalcitrance causes low yields of products and accumulation of undigested biomass, and thereby reduces the overall economic feasibility of biochemical production processes [5, 6]. For decades, processes known as pretreatment (because they occur *before* biological deconstruction) have been widely studied as methods to increase the accessibility of lignocellulose to biological attack, thereby improving the efficiency of subsequent bioconversion processes. Most pretreatment strategies, although effective at reducing recalcitrance, face challenges related to cost, operational robustness, chemical recycle, and/or production of compounds that inhibit processing [7–11]. Processes that address these challenges are critical for greater use of lignocellulosic feedstocks in a range of biofuel and bioproduct processes.

Paye et al. [12] introduced a novel process termed cotreatment (reducing recalcitrance *during* fermentation) to improve lignocellulosic biomass utilization in pure-culture systems with *Clostridium thermocellum* [13]. Mechanical cotreatment is similar to “chewing the cud”, a key part of the ruminant animal strategy for digesting lignocellulosic grasses and other forages. When chewing cud, ruminants regurgitate and masticate partially digested biomass in a cyclical manner to improve digestibility. In a ruminant this cycle of repeated fermentation (in the rumen) and mechanical disruption (in the mouth) takes place many times before the material is completely digested. The cow is estimated to use about 1% of the metabolizable energy in its feed for the cud-chewing processes, while the cow’s efficiency of biomass conversion to energy can be as high as 72% [14]. In their paper, Paye et al. [12] milled partially fermented lignocellulose and then subjected it to a second round of inoculation and fermentation, thereby employing a ferment–mill–ferment approach to investigate the cotreatment strategy. Milling after a period of fermentation presumably causes the already weakened, partially digested plant cell wall to open up further and expose more of the cellulose fibers to biological attack. In a subsequent study, Balch et al. [15] reported a significant improvement in biomass digestibility and solubilization of structural sugars in lignocellulose by the incorporation of continuous in situ ball milling during *C. thermocellum* fermentation.

Previous studies by several other research groups have observed similar results in undefined mixed-culture contexts since the early 2000s, applying some form of treatment to extract additional biomass energy from partially digested material [16–29]. Some of these studies continued to use the term pretreatment even though the biomass undergoing the treatment had been previously broken down in some fashion (e.g., manure that has already been partially digested by livestock, or biomass that has been partially digested inside an anaerobic digester). Some studies employed these additional breakdown strategies primarily for sludge dewatering, with enhanced digestion coming as a bonus [18, 20]. Other studies used the terms treatment, post-treatment, inter-treatment and digestate disintegration [23–26]. This terminology is important to the history of this innovation, as several of these prior mixed-culture studies observed that non-biological treatment may be more effective after the initial digestion rather than before digestion. [27–29].

Among studies investigating various methods to reduce biomass recalcitrance and enhance anaerobic digestion, some focused specifically on mechanical milling/maceration and reported improved biogas yields [21, 26–28]. The different mechanical milling technologies and configurations previously reported varied in their impact on the particle size reduction as well as increase in surface area, porosity, and methane yield. For example, Lindner et al. [30] reported increased methane yields and rate of biogas production, and a decrease in particle size when a range of anaerobic digestates were subjected to mechanical disruption for various milling durations using a ball mill.

In the current study we apply ball milling, a cotreatment strategy previously used for pure-culture fermentations [12, 14, 30, 31], to evaluate the impacts on mixed-culture anaerobic digestion of switchgrass, a recalcitrant lignocellulosic feedstock. One key difference from the prior pure-culture cotreatment studies is that in the current study there was no reinoculation in the second fermentation phase. In addition to measuring the effects of cotreatment on biomass degradation, the energy required for the lab-scale vibratory ball mill was also measured.

The alternating disruption of biomass by biological and mechanical treatment provides the potential for biological catalysts (such as microbes or enzymes) to access plant fibers more effectively, thereby enhancing biomass degradation. This strategy offers potential for development of milling-enhanced fermentations without addition of harsh pretreatment chemicals that often generate inhibitory by-products. This study aims to demonstrate proof-of-concept and advance understanding of cotreatment-assisted mixed-culture fermentations by using a mechanical ball-mill on partially digested switchgrass.

## Results

Partially digested switchgrass effluent from a 30-day retention time semi-continuous mesophilic anaerobic digester was collected and subsequently subjected to ball milling for various milling durations followed by a second fermentation post-milling. The solids content of the fermented and stored effluent was 43.65 g volatile solids (VS) L^−1^, considerably lower than the theoretical initial loading of 55.0 g (VS) L^−1^ because of solids degradation during the 30-day retention time of the first stage of anaerobic digestion and during the several weeks of unmixed storage it took to accumulate sufficient material for the milling experiment. Various measurements were conducted before milling, immediately after milling (indicated as ‘Day 0’) and after the second fermentation post-milling (indicated as ‘Day 18’) on the solid and liquid fraction of the material as well as the biogas produced. These include structural sugars, biogas composition and volume, total and volatile solids, particle size distribution, volatile fatty acids and ethanol concentration, and milling energy consumption.

### Structural sugar solubilization

The partially fermented material from the first fermentation and incubated storage before cotreatment had already solubilized 36.4 ± 8.1% of the sugar monomers (glucose, xylose, arabinose, and galactose) present in the structural carbohydrates in the original undigested switchgrass. In the unmilled controls, there was additional solubilization of around 2.8 ± 9.7% of those structural sugars during the second fermentation, which was not a significant improvement when compared to the partially digested material from the first fermentation (the starting material). The large standard deviations have been attributed to challenges securing representative samples from the heterogeneous lignocellulosic matrix, possible outliers (none were excluded from these results), and limited number of replicates (triplicates) per condition. Compared to the unmilled controls, the milled samples had a 4–13% increase in solubilization of structural sugars by the end of the second fermentation, and this increase was statistically significant (α = 0.1) above the 2-min milling duration.

In the complex biochemistry of undefined mixed-culture anaerobic digestion, other biomass components including proteins and lipids are also decomposed. Solublization of proteins and lipids was not directly measured because the microbiome produces as well as degrades those compounds. However, these other high energy compounds also contributed to the biogas formation discussed below.

### Biogas production

During the second fermentation period cotreatment resulted in a statistically significant increase (α = 0.1) in production of biogas (methane and carbon dioxide) relative to the unmilled control. There was also a strong trend of increasing gas production with increasing milling duration (Fig. 2A). The rate of increase in biogas production was most apparent between day 2 and 7 after cotreatment (Fig. 2B). After day 7, the rate of gas production became nearly constant across all treatments and the control, with the 10-min milled samples continuing to present a slightly higher degradation rate than the other treatments and the control throughout the rest of the second fermentation period. The CH_4_ and CO_2_ composition of the biogas at the end of the second fermentation is presented in Supplementary Fig. S1, with the methane concentrations ranging from 44% to 55.5% and the balance carbon dioxide, which is in the normal range for mixed-culture methanogenesis.

### Volatile fatty acids and volatile solids

**Fig. 1:**
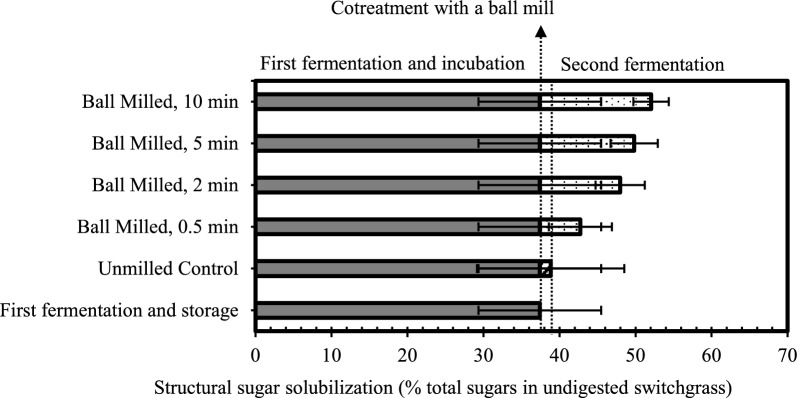
Biomass structural sugars solubilized as a percentage of the total structural sugars originally present in the undigested switchgrass through the first stage fermentation and storage, cotreatment and the second stage fermentation. The data presented are averages of triplicate reactors per condition, with error bars representing one standard deviation

**Fig. 2:**
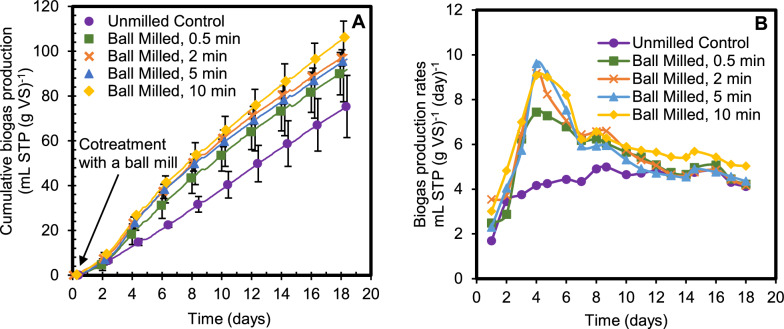
Biogas production during the second fermentation after cotreatment with a vibratory ball mill represented as **A** cumulative gas production, **B** gas production rates, both represented per gram volatile solids (VS) of partially digested switchgrass. Feedstock for this second fermentation had previously been extracted from a semi-continuous reactor with a 30-day retention time. Results presented are from triplicate reactors with error bars representing one standard deviation

Volatile fatty acids (VFAs) were measured as a potential coproduct along with biogas and sugar solubilization. VFAs can dominate the product portfolio in mixed-culture anaerobic fermentations if methanogens are inhibited, but in this case that did not occur, consistent with continued biogas production (Fig. 2) comprising more than 50% methane (Fig. S1). By the end of the 18-day second fermentation, acetic acid was found at about 0.5 mM or lower and all the other VFAs were below detection limits or absent, and therefore are not reported. These two lines of evidence (> 50% methane in the biogas and low concentrations of VFAs) indicate that methanogens were not inhibited by even the most intensive milling cotreatment tested. Comparing sugar solubilization (Fig. 1) with biogas production (Fig. 2.) in all cases the carbon mass balance closure was greater than 90%.

### Particle size change

**Fig. 3:**
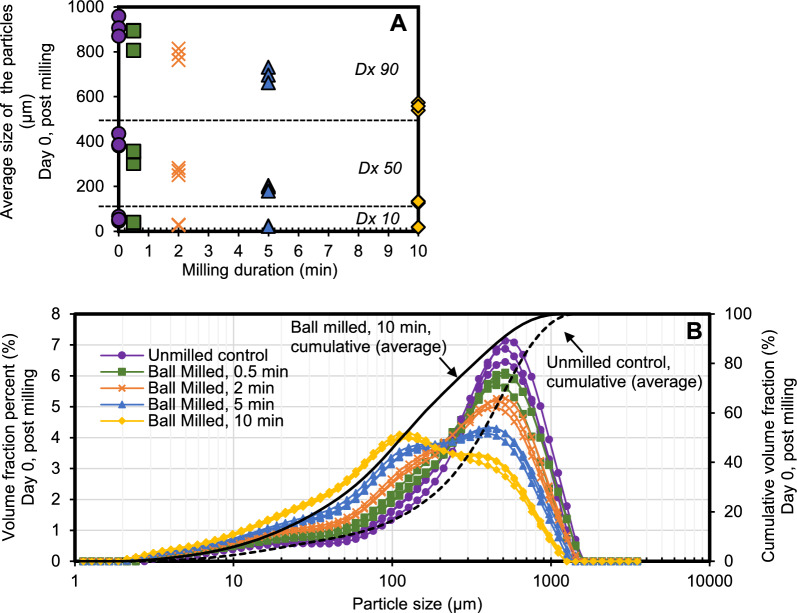
Average particle size of cotreated biomass on Day 0 after milling, represented as **A** Dx Y: Y% of particles, as a percentage of the total sample volume, are at or below this size; and B a volume-based distribution. Legend symbols for each treatment are the same for both **A** and **B**. Measurements from triplicate reactors are shown independently for each condition in both **A** and **B**. Refer to text for detailed definitions

The particle size distributions of the experimental conditions are represented in Fig. 3. Figure 3A shows Dx 90 (90% of particles are at or below the depicted particle size), Dx50 (50% of particles are at or below the depicted particle size) and Dx10 (10% of particles are at or below the depicted particle size). Figure 3B shows the same data represented as particle size distributions based on the volume fraction of the samples. A significant decrease in mean particle size was observed with increasing milling duration (Fig. 3). The particle size distribution was also measured after the second fermentation (day 18) and while a decreasing trend was observed, it was not statistically significant within each experimental condition (Supplementary Material, Fig S3).

### Energy consumed for cotreatment

**Fig. 4:**
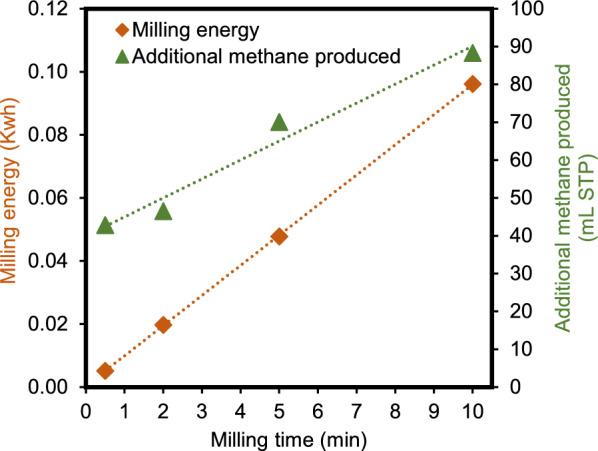
Milling energy required for cotreatment of partially digested anaerobic digestate at for various time points—0.5, 2, 5, 10 min (orange diamond) and additional methane produced compared to the unmilled control (for anaerobic digestion of 200-ml cultures containing approximately 35 g/L of partially digested switchgrass)

The additional milling energy required for longer durations of cotreatment generated diminishing amounts of additional methane (Fig. 4). Furthermore, the impact of milling in decreasing particle size, while significant as milling time is extended, also realized diminishing returns to that energy investment (Fig. 3). The total methane produced during stage two increased with milling duration, ranging from a 14% (0.5 min. milling time) to 20% (10 min. milling time) (Fig. S1), while the Energy Return on Energy Invested (EROI) decreased from 0.090 to 0.010^.^ for 0.5 and 10 min milling, respectively. While even the shortest milling duration tested had an EROI <  < 1, the shortest duration was nearly ten times as energy efficient as the longest duration tested. It is also important to note that bench-scale results are not representative of industrial milling equipment as is further discussed below. Analytical measurements showed that significant amounts of unconsumed volatile solids and structural sugars remained after the second fermentation, even for higher cotreatment milling durations. That observation, as well as the substantial amount of additional methane produced during the shortest milling time tested, suggest that repeated shorter bursts of milling between additional fermentation periods may provide many of the same benefits with a lower milling energy consumption. The power consumed by the mill was found to be consistent across all the conditions at 0.23–0.25 kW** (Supplementary Material Fig. S5).**

## Discussion

The difference between solubilization of control and cotreated samples indicates that while the mixed cultures continue to degrade biomass, extending digestion beyond 30 days without cotreatment appears to be slow and inefficient, likely due to the inaccessibility of the remaining biomass. The additional solubilization made possible through the application of cotreatment milling during fermentation is illustrated in Figs. 1 and 2.

Increased gas production relative to the control suggests that cotreatment increased accessibility of the recalcitrant fraction of the biomass to biological catalysts. Evidence of improved biomass consumption through milling has been reported in traditional mechanical pretreatment studies, where this improvement was attributed to decreases in cellulose crystallinity, particle size, and fiber thickness as well as increases in surface area and porosity [33, 34]. Barakat et al. (2013) [33] described coupling of physiochemical pretreatments with mechanical pretreatments, with the first stage causing significant biomass breakdown that drastically improved biomass sugar yield and reduced the milling energy required for the second stage. Barakat et al.’s (2013) [36] review also reported an increase in surface area and decrease in crystallinity of certain plant materials particularly through ball milling. In this study, it is likely that cotreatment provided some of the same benefits reported in these combined pretreatment studies.

Since shorter milling durations achieved statistically similar improvements in gas production as longer milling durations, shorter milling durations may present an opportunity to enhance biogas production while conserving milling energy. If a single round of ball milling only opens the plant fibers and/or decreases cellulose crystallinity to a certain degree, allowing some access for biological catalysts (enzymes or microorganisms) but not complete access, a second round of milling may offer similar benefits. Therefore, as with the chewing of cud by ruminants, fermentation with repeated short durations of milling may have a higher impact in opening up access to biomass for further degradation and be more energy efficient for running the mill.

Volatile solids consumed was also measured and showed similar trends to sugar solubilization and biogas production of increased solubilization with increased cotreatment time (Supplementary Material Fig. S2). Undigested volatile solids including structural carbohydrates (Fig. 1) continue to remain even after the second fermentation, but the gas production rates for control and cotreated samples seem to converge by day 18 (Fig. 3b). This suggests that the remaining biomass has once again become recalcitrant and inaccessible, thereby slowing the rate of digestion. Additional rounds of cotreatment may encourage further solubilization.

Although there is a significant decrease in mean particle size with increased cotreatment time, the gas production rates remain similar, supporting the hypothesis of Hartmann et al. [27] that it is recalcitrant biomass accessibility rather than particle size itself that influences biomass solubilization. Particle size is one of the factors that impacts biomass accessibility so these two factors may well interact. Though not statistically significant, there was a trend of slightly reduced particle sizes during the second fermentation, likely indicative of microbial degradation and added microbial biomass (Supplementary Fig. S3 and S4).

In the benchtop unit used in this study, operational limitations and materials handling constraints at small scale resulted in the processing of a relatively small amount of biomass diluted in a much larger mass of water, with steel balls in a thick-walled metal mill having much larger mass than the water and biomass combined. All this mass was accelerated and decelerated repeatedly for the mill to function, thereby increasing the energy required.

While ball mills are known to be energy intensive, their milling energy requirements vary significantly depending on the configuration, scale, type of biomass, processing times and when used in combination with other milling technologies [33–36]. Commercial ball mills used for large-scale operations, including for powders and minerals, have a much higher ratio of substrate to ball mill mass, and are thus likely more energy efficient. In addition to a higher solids loading of biomass, other design modifications that might be appropriate for commercial cotreatment include a higher volume to surface ratio (larger diameter balls), a larger milling chamber, and continuous flow of biomass through the mill [35].

Alternatively, a more energy efficient milling technology than ball milling could be evaluated for cotreatment. Da Silva et al. [34] reported that while there are many factors that influence lignocellulose degradation, ball milling seemed to primarily reduce cellulose crystallinity and particle size while the wet disk milling decreased fiber length and thickness, and affected accessible surface area of the biomass. Furthermore, the two mills had varied impacts on different types of biomass, sugarcane or bagasse, thereby indicating that the structure of the biomass may play an important part in determining which type of cotreatment mill is best for different feedstocks or processes.

## Conclusions

The multiple measurements reported in this study demonstrate that cotreatment for mixed-culture anaerobic fermentation systems can enhance biomass degradation and sugar solubilization, as has been observed for pure cultures [12, 15]. The results were also consistent with previously reported literature in undefined mixed-culture systems, with a general increase in biogas production after ball milling, particularly at higher milling durations [30]. In addition, this study reports that the most significant impact of cotreatment milling occurs within the first couple minutes of milling. The disproportionate advantages of short-term milling with respect to both particle size reduction and energy return on energy invested provide a basis for further studies. In addition to reducing milling time per iteration, potential process improvements include evaluating other milling technologies that may be more energy efficient, configuring mills in closed loop circuits for semi-continuous or continuous operation, and quantifying the contribution of the biological degradation towards reduction in subsequent milling energy consumption. Various milling treatment strategies impact a combination of physical and biochemical factors such as crystallinity, porosity, lignin content and distribution, and surface area, and may also impact which organisms are dominant in the microbiome. Given these multiple factors and the potential for interactions between milling technology and the type of biomass, further studies and scale-up analysis are needed for successful implementation of a cotreatment strategy for efficient solubilization of lignocellulose [33].

## Methods

**Fig. 5:**
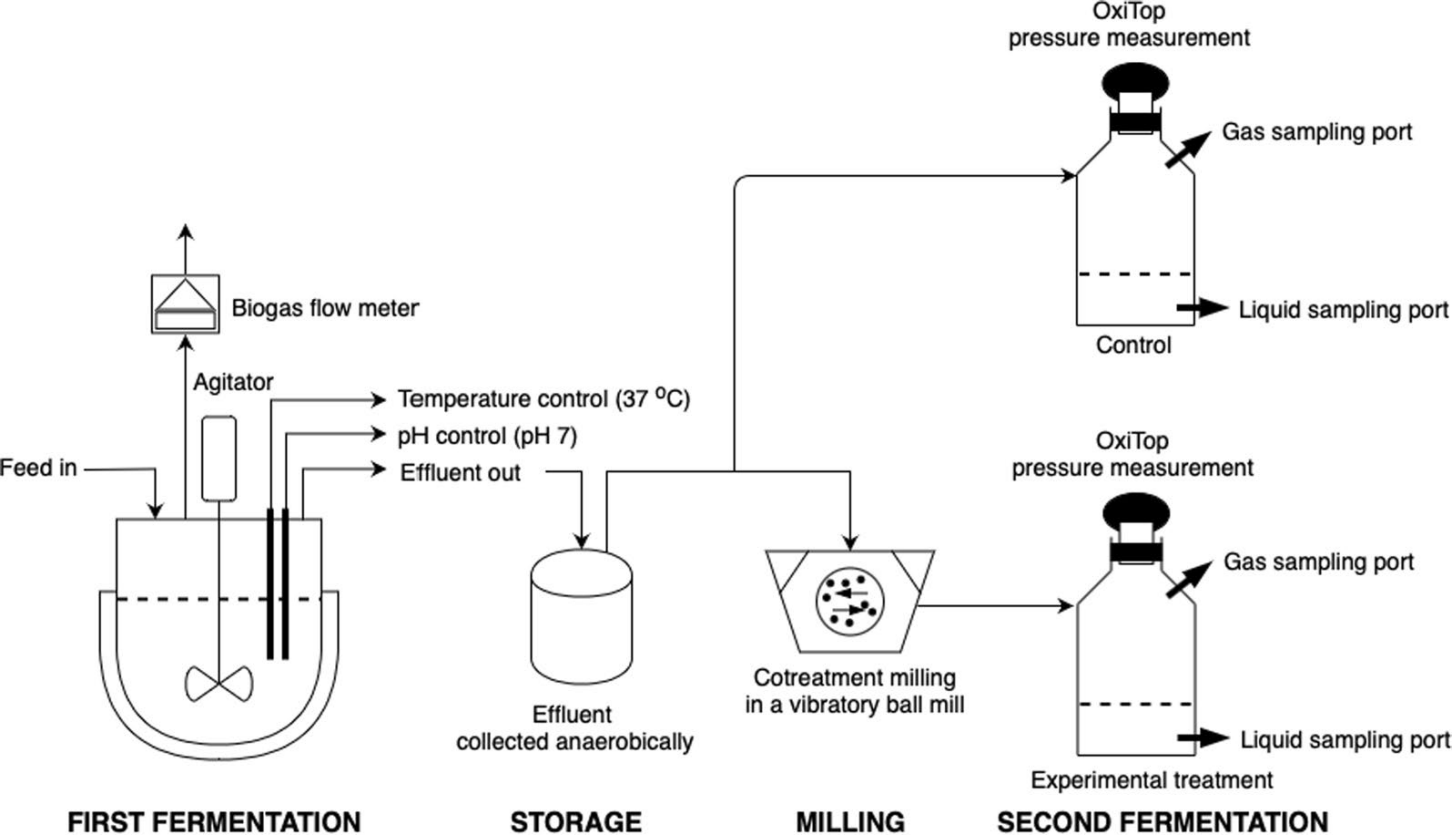
“Ferment–mill–ferment” experimental design to demonstrate cotreatment using a vibratory ball mill

The simple cotreatment strategy tested was a single round of milling after an initial fermentation that partially digested the switchgrass, followed by a second fermentation. This experimental design has been termed “ferment–mill–ferment” [12]. The partially fermented material was passed through the mill and subsequently allowed to continue to ferment in the second stage without any reinoculation as shown in Fig. 5.

### Collection of inocula

The sources of the microbiomes for inoculating the semi-continuous anaerobic digester were sewage biosolids, rumen fluid solids, and compost. The sewage biosolids were collected from the mesophilic secondary digester at The Pennsylvania State University Wastewater Treatment Plant (State College, PA, USA); the rumen fluid was collected from a fistulated cow at The Pennsylvania State University Dairy Barn (University Park, PA, USA); and the compost was collected from The Pennsylvania State University Composting Facility (State College, PA, USA).

The inocula were collected on the day of the reactor set-up, transported to the lab, and placed inside an anaerobic chamber (Coy Laboratory Products, Inc, Grass Lake, MI, USA) under a nitrogen atmosphere to minimize exposure to oxygen. The sewage biosolids and rumen fluid were separately centrifuged at 3,200 × *g* for 30 min to remove excess water, and the pellets were stored anaerobically. A small homogeneous fraction of each inoculum source was analyzed for total solids (TS) and volatile solids (VS) content to allow for proportional blending on a VS basis.

### Lignocellulosic biomass

Oven-dried senescent switchgrass (Hobbs Shawnee variety, Ernst Biomass LLC, Meadville PA) was milled to ≤ 1 mm particle size using a knife mill (Thomas Wiley mill, Thomas Scientific, Swedesboro, NJ, USA) and stored in a cool dry room. The moisture content of the material was determined to be 8.7% (wet basis), ash content was 8.0% (dry basis) and the structural sugar content was 63.2% (dry basis). No additional pretreatment or autoclaving was conducted on the switchgrass material before use.

### Fermentation medium

An enriched anaerobic medium with inorganic nitrogen, phosphorus, micronutrients, and vitamins was added to eliminate potential medium constraints on the growth and maintenance of the microbiome during anaerobic digestion. The anaerobic medium composition was based on Angelidaki et al. (2009) and was modified to enable the efficient dissolution and bioavailability of all components (Supplementary Material Table S1) [37]. Additional carbon sources were eliminated except vitamins and EDTA so that the system was dependent on the senescent switchgrass as its sole carbon source.

### Set-up and operation of first stage semi-continuous anaerobic digester

The above-mentioned inoculum sources (sewage biosolids, rumen fluid, and compost) were combined in 1:1:1 ratio (g VS) based on the previously determined VS measurements. This combined inoculum mix was then blended in the medium with the switchgrass at 1:2 ratio (g VS of inoculum mix: switchgrass) to achieve a total initial VS of 20 g L^−1^. Solids loading of the semi-continuous 4-L continuously stirred tank reactor was slowly ramped up to 1.84 g (VS) L^−1^ day^−1^ with a retention time of 30 days, resulting in a theoretical solid loading of 55 g (VS) L^−1^. This was achieved by replacing 3.33% (133 ml) of the volume once a day, 7 days a week with fresh medium and switchgrass. This slow ramp-up enabled the reactor to reach steady state without an acid upset using unpretreated switchgrass as the primary carbon source. The effluent from this complete mix, semi-continuous digester was collected immediately prior to the daily feeding and stored anaerobically at 37 °C in an incubator over several weeks to accumulate sufficient material for the milling experiment. This accumulated digestate was thoroughly mixed prior to milling, with the same mixture was used for the unmilled control so that any effects of accumulation and storage were consistent across all treatments and the control.

### Cotreatment: milling of anaerobic digestate from the first stage

Once enough effluent material for the milling trials was collected, it was mixed with sodium bicarbonate buffer to a final concentration of 50 mM and a solids concentration of approximately 35 g (VS) L^−1^. Aliquots of this partially fermented digestate were then milled anaerobically for predetermined times—0.5, 2, 5, and 10 min—using a vibratory ball mill with four 6 mm, four 8 mm, four 10 mm, and two 12 mm stainless steel balls (MSK-SFM-3, MTI Corporation, Richmond, CA, USA).

The energy consumed by the mill during cotreatment was measured using a data logger (ELETE Pro, Dent Instruments, Bend, OR, USA). The milling was conducted entirely under anaerobic conditions inside the anaerobic chamber. For each milling time treatment, 200 ml aliquots of the milled effluent were then loaded in triplicate 1-L reactors for the second fermentation. No spinning down or reinoculation of the material was conducted. Unmilled effluent material was also loaded in triplicate 1-L reactors to be used as the control, providing a baseline estimate of the continued solubilization of the biomass if cotreatment had not occurred.

### Second stage digestion: batch fermentation tests

The fermentation after milling was modeled after the BioMethane Potential (BMP) test used to characterize bioconversion rates and methane yield under anaerobic conditions but was modified to specifically assess cotreatment impacts [37]. The reactors used for this second fermentation were 1-L Schott bottles modified with side ports for gas and liquid sample collection. No additional inoculum or media were added post-milling and the second fermentation continued solely with the microbes, substrate, and nutrients already present in the material. This avoided possible interferences that may otherwise mask the observed cotreatment impacts. The bottles were capped with OxiTop® pressure sensors (Xylem Analytics Germany Sales GmbH & Co. KG, WTW, Weilheim, Germany) to record gas production over time. A negative control containing just 1X sodium bicarbonate buffer was used in duplicate to account for headspace pressure fluctuations inside the incubator.

The bottles were placed in an incubator at 37 °C and left to ferment for 18 days. The bottles were stirred manually once per day and vented as needed, always prior to when the OxiTops® reached their maximum pressure limit of 400 hPa. The vented gas was collected in gas bags (Cali-5-Bond™, Calibrated Instruments Inc., McHenry, MD, USA). After the second fermentation, the bottles were vented and the material inside was used to measure various parameters described in the Analytical methods section.

### Analytical methods

Biomass structural sugars, volatile solids, volatile fatty acids, and particle size were measured immediately after milling (“Day 0”) and after the second stage fermentation (“Day 18”) from each bottle. Biogas volume was measured from the OxiTops® and biogas composition was measured on the vented gas accumulated in gas bags throughout the second fermentation.

Total and volatile solids were determined by the National Renewable Energy Laboratory (NREL) Laboratory Analytical Procedure (LAP) [38, 39]. The amount of structural sugars present in the sample was determined by quantitative saccharification (QS), a simplified version of the NREL biomass compositional analysis [40]. This process involves acid hydrolysis of the sample with 72% sulfuric acid at 30 °C for 1 h followed by autoclaving with 4% sulfuric acid [12, 15]. The monomeric sugars were measured using an ion chromatography (IC) system (IC 3000, Thermo Fisher Scientific Dionex, Sunnyvale, CA, USA) with a Carbopac™ PA20 column and an electrochemical detector with a gold electrode. The organic acid concentrations were measured using a gas chromatography system with flame ionization detector (GC-FID) system (Shimadzu GC-2010, Shimadzu Scientific Instruments, Columbia, MD, USA) and a Stabilwax-DA column.

The biogas evolved was collected in gas bags and analyzed for methane, hydrogen, carbon dioxide, and oxygen concentrations using a second gas chromatography system with thermal conductivity detector (Multiple Gas Analyzer GC #5, SRI Instruments, Torrance, CA, USA), argon as the carrier gas, and a Molecular Sieve and Hayesep columns.

The particle size distribution of the samples before and after the second fermentation was measured using a laser diffraction particle size analyzer (Mastersizer 3000™, Malvern PANalytical Ltd, Malvern, WR, UK) at the Materials Characterization Laboratory, The Pennsylvania State University (University Park, PA). The measurements were conducted in triplicate with a medium sized liquid module with deionized water as the dispersant, reference refractive index as cellulose, and particle shape set to ‘irregular’. The samples were homogenized and dispersed using a transfer pipette to achieve an obscurity between 9%-15%.

### Statistical analyses and calculations

A randomized block design was used for the experiments with time as the block variable due to the long duration (12 – 14 h) required for set up. One control and one of each experimental treatment was assigned within each block. Statistical tests included ANOVA statistical test with blocking, Bonferroni correction for multiple comparisons with two-sided (alternative hypothesis: means of experimental treatments ≠ means of control) and Dunnett’s one-sided lower bound test (alternative one-directional hypothesis: mean of experimental treatment > mean of control); each test considered a Type I error (α) of 0.1.

The percentage biomass structural sugars solubilized was calculated as follows:

1 $$\text{\% sugar solubilization}=\left(\frac{\left(\text{Sugars},\text{ Day }0\right)-\left(\text{Sugars},\text{ Day }18\right)}{\left(\text{Sugars},\text{ Day }0\right)}\right)\times 100\text{\%}.$$

The Energy Return on Energy Invested (EROI) [41] was calculated as follows:

2 $$EROI= \frac{{E}_{{CH}_{4}{cotreated}_{18}-}{E}_{{CH}_{4}{control}_{18}}}{{E}_{milling}},$$

where ${E}_{{CH}_{4}{cotreated}_{18}}$= average theoretical energy from methane generated by milling treatment replicates during the 18-day stage 2 fermentation.

${E}_{{CH}_{4}{control}_{18}}$= Average theoretical energy from methane generated in control replicates during the 18 day stage 2 fermentation.

${E}_{milling}$= Energy used for cotreatment milling.

### Supplementary Information

Supplementary Material 1.

Supplementary Material 2.

Supplementary Material 3.

## Data Availability

All data generated in this study are made available in this manuscript and supplementary materials found here: https://www.datacommons.psu.edu/commonswizard/MetadataDisplay.aspx?Dataset=6366.

## Abbreviations

DM: Dry matter
PSD: Particle size distribution
STP: Standard temperature and pressure
VS: Volatile solids
