# Supplementary material for: Enhancing anaerobic digestion of lignocellulosic biomass by mechanical cotreatment
Source: Biotechnol Biofuels Bioprod. 2024 Jun 3;17:76. doi: 10.1186/s13068-024-02521-5 (PMC11149370; doi:10.1186/s13068-024-02521-5)
Supplement: Supplementary file 1 — Supplementary Material 1. [file 13068_2024_2521_MOESM1_ESM.docx]

**Supplementary material**

**Table S1.** A modified version of the anaerobic medium recommended by Angelidaki et al. [30]. The recipe was modified to prevent reaction and precipitation of some compounds when heated in the autoclave.

| 1L of medium was prepared by mixing stock solution A (10 mL), solution B (2 mL), solution C (1 mL), and solution D (1 mL) and 986 mL of autoclaved distilled water. Concentrations are expressed per L of stock solution. | | |
| --- | --- | --- |
| Solution A (autoclaved) | Ammonium chloride (NH_4_Cl) | 100g L^-1^ |
|  | Sodium chloride (NaCl) | 10 g L^-1^ |
|  | Magnesium chloride (MgCl_2_.6H_2_O) | 10 g L^-1^ |
|  | Calcium chloride (CaCl_2_.2H_2_O) | 5 g L^-1^ |
| Solution B (filter sterilized) | Potassium monophosphate (K_2_HPO_4_.3H_2_O) | 200 g L^-1^ |
| Solution C (autoclaved)  (EDTA was added first and the pH was brought up to 8 using sodium hydroxide pellets. The rest of the components were added and the pH was adjusted to 7 using hydrochloric acid) | Ferrous chloride (FeCl_2_.4H_2_O) | 2 g L^-1^ |
|  | Boric acid (H_3_BO_3_) | 0.05 g L^-1^ |
|  | Zinc chloride (ZnCl_2_) | 0.05 g L^-1^ |
|  | Cupric chloride (CuCl_2_.2H_2_O) | 0.038 g L^-1^ |
|  | Manganese chloride (MnCl_2_.4H_2_O) | 0.05 g L^-1^ |
|  | Ammonium molybdate (NH_4_)6Mo_7_O_24_.4H_2_O | 0.05 g L^-1^ |
|  | Aluminum chloride (AlCl_3_) | 0.05 g L^-1^ |
|  | Cobalt chloride (CoCl_2_.6H_2_O) | 0.05 g L^-1^ |
|  | Nickel chloride (NiCl_2_.6H_2_O) | 0.092 g L^-1^ |
|  | Hydrochloric acid (HCl) | 1 mL |
|  | Sodium selenite (Na_2_SeO_3_.5H_2_O) | 0.1 g L^-1^ |
|  | Sodium tungstate (Na_2_WO_4_.2H_2_O) | 0.1 g L^-1^ |
|  | Ethylenediaminetetraacetate (EDTA) | 0.5 g L^-1^ |
| Stock D (filter sterilized) | Biotin | 2 mg L^-1^ |
|  | Folic acid | 2 mg L^-1^ |
|  | Pyridoxine hydrochloride | 10 mg L^-1^ |
|  | Riboflavin | 5 mg L^-1^ |
|  | Thiamine hydrochloride | 5 mg L^-1^ |
|  | Cyanocobalamin | 0.1 mg L^-1^ |
|  | Nicotinic acid | 5 mg L^-1^ |
|  | p-aminobenzoic acid | 5 mg L^-1^ |
|  | Lipoic acid | 5 mg L^-1^ |
|  | Pantothenic acid | 5 mg L^-1^ |

**Table S2.** pH of the material in the batch fermentation bottles on day 0 and day 18 of the second fermentation. Measurements from each triplicate reactor is presented independently.

| Sample (after buffer addition) | pH |
| --- | --- |
| Starting material (day 0) | 7.3 |
| Day 18 |  |
| Block A  Ball milled 0.5 min  Ball milled 2 min  Ball milled 5 min  Ball milled 10 min  Unmilled Control | 7.18  7.23  7.44  7.19  7.58 |
| Block B  Ball milled 0.5 min  Ball milled 2 min  Ball milled 5 min  Ball milled 10 min  Unmilled Control | 7.24  7.49  7.2  7.15  7.48 |
| Block C  Ball milled 0.5 min  Ball milled 2 min  Ball milled 5 min  Ball milled 10 min  Unmilled Control | 7.37  7.41  7.4  7.23  7.44 |

**Fig. S1.** Biogas production and composition after second stage fermentation following ball milling, compared to the unmilled control (* indicates average of duplicates, all other conditions are averages of triplicates, error bars indicate one standard deviation).

**Fig. S2.** Percentage of volatile solids of partially digested switchgrass consumed during the second fermentation after cotreatment with the vibratory ball mill. Data represent the average of triplicate reactors for each condition, with error bars representing one standard deviation.

**Fig. S3.** Average particle size of cotreated biomass at the end of the second fermentation period (day 18) represented as (A) Dx Y: Y% of particles, as a percentage of the total sample volume, are at or below this size (B) a volume-based distribution. Legend symbols for each treatment are the same for both (A) and (B). Measurements from the triplicate reactors of each condition are shown independently.

**Fig. S4.** Comparison of particle size distribution for various cotreatment conditions on day 0 and day 18. The decrease in particle size between day 0 and day 18 after cotreatment may be attributed to microbial degradation during the second fermentation as well as accumulated microbial biomass. Dx 50 represents the size of 50% of the particles as a percentage of total solid sample volume, are at or below that size. The solid lines are Dx 50 at day 0 (immediately after milling) and dashed lines are Dx 50 at day 18 after second fermentation. The data represents averages of triplicate reactors per condition and error bars not shown.

**Fig. S5.** Average power drawn by ball mill did not change with milling time. Results shown are averages of triplicate reactors per condition with error bars indicating one standard deviation. Power was measured every 3 seconds.
